# Effect of polyethylene glycol 20 000 on protein extraction efficiency of formalin-fixed paraffin-embedded tissues in South Africa

**DOI:** 10.4102/ajlm.v10i1.1122

**Published:** 2021-12-17

**Authors:** Sophia Rossouw, Hocine Bendou, Liam Bell, Jonathan Rigby, Alan Christoffels

**Affiliations:** 1 South African Medical Research Council Bioinformatics Unit, South African National Bioinformatics Institute, University of the Western Cape, Cape Town, South Africa; 2 Centre for Proteomic and Genomic Research, Observatory, Cape Town, South Africa; 3 Department of Anatomical Pathology, National Health Laboratory Service, Tygerberg Hospital, Stellenbosch University, Cape Town, South Africa

**Keywords:** mass spectrometry, formalin-fixed paraffin-embedded proteomics, archival tissue, protein extraction, polyethylene glycol 20 000, SP3-on-bead-digestion

## Abstract

**Background:**

Optimal protocols for efficient and reproducible protein extraction from formalin-fixed paraffin-embedded (FFPE) tissues are not yet standardised and new techniques are continually developed and improved. The effect of polyethylene glycol (PEG) 20 000 on protein extraction efficiency has not been evaluated using human FFPE colorectal cancer tissues and there is no consensus on the protein extraction solution required for efficient, reproducible extraction.

**Objective:**

The impact of PEG 20 000 on protein extraction efficiency, reproducibility and protein selection bias was evaluated using FFPE colonic tissue via liquid chromatography tandem mass spectrometry analysis.

**Methods:**

This study was conducted from August 2017 to July 2019 using human FFPE colorectal carcinoma tissues from the Anatomical Pathology department at Tygerberg Hospital in South Africa. Samples were analysed via label-free liquid chromatography tandem mass spectrometry to determine the impact of using PEG 20 000 in the protein extraction solution. Data were assessed regarding peptide and protein identifications, method efficiency, reproducibility, protein characteristics and organisation relating to gene ontology categories.

**Results:**

Polyethylene glycol 20 000 exclusion increased peptides and proteins identifications and the method was more reproducible compared to the samples processed with PEG 20 000. However, no differences were observed with regard to protein selection bias. We found that higher protein concentrations (> 10 µg) compromised the function of PEG.

**Conclusion:**

This study indicates that protocols generating high protein yields from human FFPE tissues would benefit from the exclusion of PEG 20 000 in the protein extraction solution.

## Introduction

Archival formalin-fixed paraffin-embedded (FFPE) tissue repositories are valuable resources for clinical proteomic studies; such repositories may include retrospective as well as protein biomarker discovery and validation studies.^1,2,3^ These repositories are often composed of a large variety of patient biopsy tissues, which are accompanied by their associated clinical metadata, in the form of patient medical records. The wealth of information stored in these archival FFPE tissue repositories, together with the easily accessible FFPE samples, has generated improved methods for FFPE tissue analysis in the context of genomic, proteomic and immunohistochemical studies.^1,2,3^

The development and standardisation of FFPE sample processing for mass spectrometry (MS)-based analysis to determine changes (or similarities) in the proteome composition of tumour versus healthy tissues is of great interest to clinical and translational research.^4,5^ Part of this process involves using an optimal and efficient protein extraction buffer to generate reproducible results. Studies have found that experimental factors, such as protein extraction buffer, pH, detergents, denaturants and temperature, play important roles in the final attainable protein yield from FFPE tissues.^3,6^ Other factors to consider include limited availability of clinical specimens and therefore a limited amount of starting material (tissue) available for optimising a protein extraction procedure. This places limitations on the choice of proteomics workflows (including protein extraction, protein sample enrichment, fractionation and digestion) that can be used to generate samples of suitable quality for high sensitivity liquid chromatography (LC) tandem MS analysis.^2,7^ Additional challenges faced in FFPE proteomics studies, which cannot be remedied after the fact, are pre-analytical factors that affect protein extraction efficiency and often produce variable protein yields. These may include tissue ischemic time, the composition of the fixative, fixation time (duration or range of formalin-fixation times used), as well as block age and storage conditions.^2,4,8^

During the protein extraction process, the effect of the formaldehyde fixation chemistry on the tissues poses another challenge to overcome. Due to extensive formaldehyde cross-linking between molecules, accurate and efficient protein extraction from FFPE tissues is difficult. It requires specific sample processing techniques to allow for complete breakage of cross-linking bonds, which in turn allows for proper trypsin digestion.^2,9,10,11^ For this reason multiple strategies have been employed, including the use of denaturants, detergents, precipitants and antigen retrieval. However, several aspects of the formaldehyde-protein interactions remain unresolved and are the focus of continued research in the FFPE proteomics field.

We have previously studied the effects of FFPE block age on the quality and quantity of protein extracted from FFPE tissues and also evaluated protein purification methods using LC-MS/MS analysis.^12^ However, the optimal protein extraction buffer components were not investigated. Therefore, of interest to this study are the effects of polyethylene glycol (PEG), specifically PEG 20 000, on protein extraction efficiency of human FFPE tissues using LC-MS/MS analysis, as there is no current consensus with regard to PEG usage and advantages for human FFPE tissue proteomics. Polyethylene glycol, a high molecular weight synthetic polymer, reduces non-specific protein adsorption to surfaces, such as experimental plastic-ware (micropipette tips and microcentrifuge tubes), thereby preventing subsequent protein loss.^3,13^ Polyethylene glycols also precipitate proteins through a steric exclusion mechanism, whereby they occupy most of the space in solution, thus concentrating the proteins until they exceed solubility and precipitate.^14,15,16,17^ Therefore, subsequent centrifugation may pellet the precipitated proteins^17^ and these may be lost in the sample pellets (after clarifying the protein lysates and removal of the supernatants for analysis). Polyethylene glycol also causes interference and ion signal suppression in downstream LC-MS/MS analysis, if it is not completely removed from the sample analysed.^5,13^ Removal of high concentrations of PEGs is challenging and PEG carry-over into sample fractions and LC columns is a huge problem.^14,18^ However, due to its advantages and available techniques to remove PEG before LC-MS/MS analysis, it is often used for protein extraction of FFPE tissues.^3,13^ To our knowledge, however, PEG efficacy with regard to protein extraction of human FFPE tissues has not been fully evaluated yet.

Polyethylene glycol can vary in polymer size, and for this study PEG 20 000 was chosen, because it is the most extensively used form in FFPE tissue proteomics; subsequently all references to PEG in this article are to the 20 000 form. The aim of this study was to evaluate the effects of PEG within the protein extraction buffer using label-free LC-MS/MS analysis of manually micro-dissected FFPE human colorectal carcinoma (CRC) resection samples. The sample pellets were also tested for residual protein, which was not extracted in the whole cell protein lysates (WCPLs).

## Methods

### Ethical considerations

Ethics clearance was obtained from the Health Research Ethics Committee of Stellenbosch University (ethics reference number: S17/10/203) and Biomedical Science Research Ethics Committee of the University of the Western Cape (ethics reference number: BM17/7/15). All patient specimens were anonymised before being archived for long-term storage and before they were accessed for the study. Patient consent was not required since it was a retrospective study using archival tissues.

### Formalin-fixed paraffin-embedded human colorectal carcinoma samples

This study conducted from August 2017 to July 2019, included retrospectively chosen human colorectal resection specimens acquired from the department of Anatomical Pathology at Tygerberg Hospital in Western Cape, South Africa. The specimens were preserved as FFPE blocks when the tissue was resected and archived between January 2016 and December 2017. Due to retrospective collection of the samples, the exact pre-analytical factors, such as the handling, fixation times and conditions, and storage conditions, were unknown and could not be accounted for. Table 1 shows the details of the three patient cases selected.

**TABLE 1:** Details of the three FFPE patient cases selected for analysis at the South African National Bioinformatics Institute, University of the Western Cape, Bellville, South Africa, from August 2017 to July 2019.

Meta data tags	Patient 1	Patient 2	Patient 3
Block year	2017	2016	2016
Patient age (years)	60	47	60
Gender	Female	Male	Male
Diagnosis	Adenocarcinoma	Adenocarcinoma	Adenocarcinoma
Grade	Low-grade	High-grade	Low-grade
Stage	IIIB	IIIB	IIA
Location	Right colon	Right colon	Right colon

**FIGURE 1:**
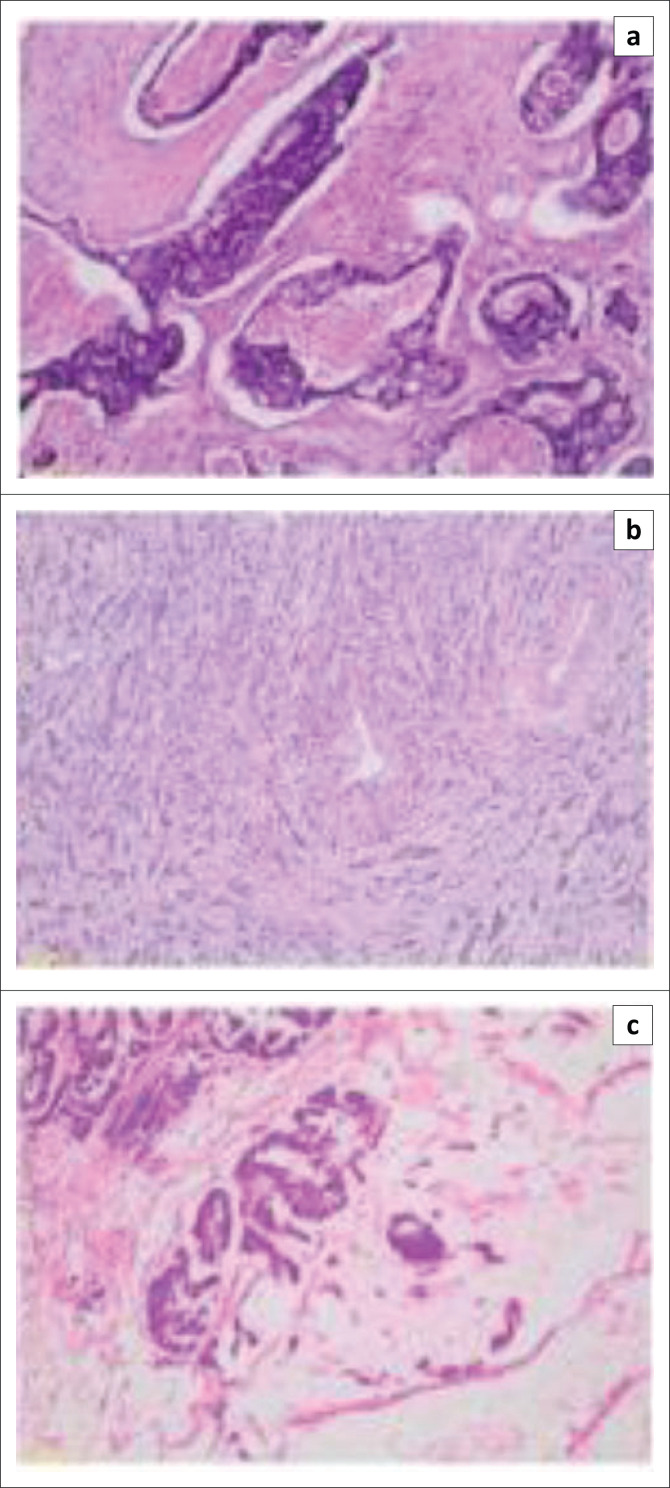
Colon adenocarcinoma tissue specimens analysed at the South African National Bioinformatics Institute, University of the Western Cape, Bellville, South Africa, from August 2017 to July 2019. Microscopic images of haematoxylin and eosin-stained colon tissue sections of patient resection specimens analysed in this study: patient 1 (a), patient 2 (b) and patient 3 (c) at 100× magnification.

To ensure tissue quality and comparability, a pathologist reviewed the patient tissue sections after haematoxylin and eosin staining to select only specimens that had carcinomas with more than 90% viable tumour nuclei (Figure 1). Patient samples were also classified and diagnosed with low-grade or high-grade colorectal carcinoma after haematoxylin and eosin staining.

### Protein extraction and purification

To overcome the effects of formaldehyde cross-linking, we opted to combine protein extraction techniques that employed the use of antigen retrieval, strong detergent concentration, as well as a synthetic polymer for protein precipitation (PEG 20 000). For protein purification before LC-MS/MS analysis, we used the Single-Pot Solid-Phase-enhanced Sample Preparation (SP3)^19,20^ method, which ensures minimal sample loss during processing and was also found to be highly sensitive, therefore requiring less starting material (tissue).^12,19,20^

**FIGURE 2:**
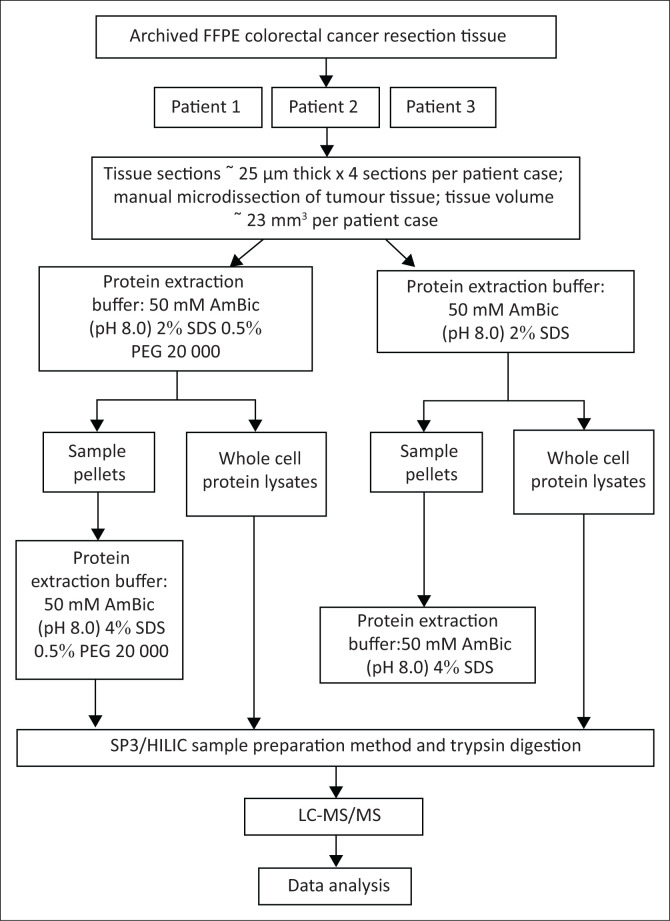
Summarised workflow and experimental design followed at the South African National Bioinformatics Institute, University of the Western Cape, Bellville, South Africa, from August 2017 to July 2019. FFPE colorectal carcinoma tissue from three patients were cut at 25 µm thickness and tumour areas were manually micro-dissected for analysis. From each patient FFPE block, 4 tissue sections (each 25 µm thick and equivalent to approximately 23 mm^3^ tissue) were used per experimental sample. Protein extraction buffer, with or without the addition of PEG, was used to extract protein. Sample pellets were analysed for residual protein by further protein extraction (using 4% SDS), followed by protein quantification and subsequent sample processing (for LC-MS/MS analysis) by the HILIC/SP3 method. WCPLs from each patient were quantified and processed by the HILIC/SP3 sample preparation method, followed by MS analysis. The mass spectra generated were then analysed during the data analysis phase.

The equivalence of 23 mm^3^ of manually micro-dissected FFPE tumour tissue was cut and processed for each patient case (Figure 2). Protein was extracted using a solution that consisted of 50 mM of ammonium bicarbonate (pH 8.0) and 2% sodium dodecyl sulphate (SDS) and either with or without the addition of 0.5% PEG. To further determine protein extraction buffer efficiency, the sample pellets were also assessed for residual proteins that were not extracted in the initial extraction. In total, 12 samples were analysed, including the WCPLs as well as the sample pellets (Figure 2).

The method used for sample processing, protein extraction and protein yield quantification was modified from the protocols used by Scicchitano^21^ and Wiśniewski^22^ and previously described in more detail by Rossouw.^12^ Both WCPLs and sample pellets were subsequently processed by the hydrophilic interaction liquid chromatography (HILIC)/SP3 magnetic bead digestion method,^19^ before LC-MS/MS analysis (Figure 2).

The MagReSyn® (ReSyn Biosciences, Edenvale, Gauteng, South Africa) HILIC/SP3 method (using on-bead digestion) was used for protein purification and tryptic digestion (peptide generation) prior to LC-MS/MS analysis. The method was modified from the protocol used by Hughes^19^ and previously described in more detail by Rossouw.^12^

### Mass spectrometry analysis

Mass spectrometry analysis of each sample’s peptides was performed using the Q-Exactive quadrupole-Orbitrap (Thermo Fisher Scientific, Waltham, Massachusetts, United States), which was coupled with a Dionex Ultimate 3000 nano-UPLC system as described before by Rossouw.^12^ Using Xcalibur^TM^ (version 4.2) (Thermo Fisher, Waltham, Massachusetts, United States), spectral data was collected in a data-dependent manner and details are shown in Supplementary document – Table S1.

### Identification of peptides and proteins

**FIGURE 3:**
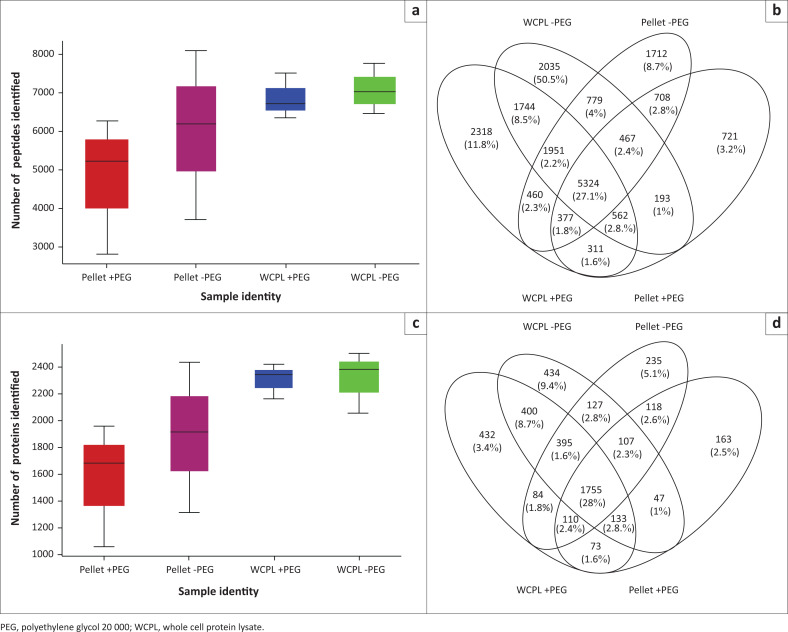
Numbers of identified peptides and proteins from WCPLs and pellets, compared between different protein extraction buffers with or without addition of PEG at the South African National Bioinformatics Institute, University of the Western Cape, Bellville, South Africa, from August 2017 to July 2019. (a) Box and whiskers plot showing the number of peptides identified (for all three patient samples) per condition – Pellet with PEG (4% SDS), Pellet without PEG (4% SDS), WCPL with PEG (2% SDS), WCPL without PEG (2% SDS). (b) Venn diagram depicting the distribution of identified peptides (for all three patient cases) among all conditions. (c) Box and whiskers plot showing the number of proteins identified (for all three patient samples) per condition. (d) Venn diagram depicting the distribution of identified proteins (individual and protein groups) (for all three patient cases) among all conditions. (–PEG) refers to protein extracted without PEG and (+PEG) refers to protein extracted with PEG. Red boxplots refer to pellet samples extracted with PEG; Purple boxplots refer to pellet samples extracted without PEG; Blue boxplots refer to WCPL samples extracted with PEG; Green boxplots refer to WCPL samples extracted without PEG.

The raw spectral data were converted into ‘mascot generic format’ (Matrix Science, London, United Kingdom), which is a standard format used for tandem MS data that converts the raw data into a simpler format for subsequent database searches, using msConvert (ProteoWizard, Palo Alto, California, United States).^23^ X!Tandem (version 2015.12.15.2)^24^ (The Global Proteome Machine Organization, Winnipeg, Manitoba, Canada), MS Amanda (version 2.0.0.9706)^25^ (Protein Chemistry Facility IMP/IMBA/GMI, Vienna, Austria), and MS-GF+ (version 2018.04.09)^26^ (Pacific Northwest National Laboratory, Richland, Washington, United States) were used to identify peak lists from MS/MS spectra.^12^ SearchGUI (version 3.3.3)^27^ (Computational Omics and Systems Biology Group, Ghent University, Gent-Zwijnaarde, Belgium) was used to allow for simultaneous searches. A concatenated target-decoy^28^ version of the *Homo sapiens* (73101, > 99.9%), *Sus scrofa* (1, < 0.1%) complement of the UniProtKB^29^ reference proteome (UP000005640; 9606-*Homo sapiens*) (version downloaded on 29/10/2018) was used for protein identifications. SearchGUI generated the decoy sequences. The identification settings are shown in Table 2 and the certificate of analysis lists all algorithms settings used and validation thresholds (Supplementary document 3 – File S1). PeptideShaker (version 1.16.31)^30^ (Computational Omics and Systems Biology Group, Ghent University, Gent-Zwijnaarde, Belgium) was used to infer peptide and protein identifications from spectrum identification data and validated at 1% false discovery rate estimated using the decoy hit distribution. D-score^31^ and phosphoRS score^32^ (threshold of 95.0 as implemented in the compomics utilities package^33^) were used to score post-translational modification localisations. Adequate or acceptable reproducibility or reliability, as it pertains to the results (including Figure 3a and 3c), was defined as the observable extent (measured, for example, as the standard deviation) of stability within measured data points when measurements are repeated under similar experimental conditions.

**TABLE 2:** Peptide and protein identification settings at the South African National Bioinformatics Institute, University of the Western Cape, Bellville, South Africa, from August 2017 to July 2019.

Parameter	Settings
Trypsin digestion	Specific, maximum of 2 missed cleavages
MS1 tolerance	10.0 ppm
MS2 tolerance	0.02 Da
Fixed modifications	Methylthio of C (+45.987721 Da)
Variable modifications	Oxidation of M (+15.994915 Da), Deamidation of N and Q (+0.984016 Da)
Fixed modifications (refinement procedure)	Methylthio of C (+45.987721 Da)
Variable modifications (refinement procedure)	Acetylation of protein N-term (+42.010565 Da), Pyrolidone from E (--18.010565 Da), Pyrolidone from Q (--17.026549 Da), Pyrolidone from carbamidomethylated C (--17.026549 Da)

MS1, first stage of mass spectrometry; MS2, second stage of mass spectrometry; ppm, parts per million; Da, Dalton; C, cysteine; M, methionine; N, asparagine; Q, glutamine; N-term, N-terminal; E, glutamic acid.

### Data analysis

Data were analysed and graphically visualised and displayed using Pandas, NumPy and Matplotlib Python packages (Python Software Foundation, Wilmington, Delaware, United States), as well as Microsoft Excel (Microsoft Corporation, Redmond, Washington, United States).

The merged lists of either peptide sequences or protein accession numbers (individual as well as protein groups) identified in each sample group and experimental condition were processed using Venny (version 2.1.0)^34^ (BioinfoGP Service, Universidad Autónoma de Madrid, Madrid, Spain), which calculated and visually displayed the percentage overlap.

To determine the qualitative reproducibility of each experimental condition, the peptide identification overlap (Supplementary document – Figure S1) was computed using the peptide sequences identified for each sample from the data set (regardless of peptide abundance). From these results, the physicochemical properties of the peptides (unique as well as shared) for all conditions were assessed for each patient (Supplementary document – File S2).

Spectrum counting abundance indexes were estimated using the Normalised Spectrum Abundance Factor^35^ as generated by the PeptideShaker software.^27,36^ The Normalised Spectrum Abundance Factor values were normalised to facilitate comparisons and then used to calculate the Pearson’s correlation coefficient, for each pair of experimental conditions compared with regard to differential protein abundance, to determine the level of correlation between samples (Supplementary document – Figure S2).

ProPAS (version 1.1)^37^ (State Key Laboratory of Proteomics, Beijing Institute of Radiation Medicine, Beijing, China) was used to calculate the physicochemical properties (hydropathicity [Kyte and Doolittle scale],^38^ molecular weight and isoelectric point) of identified peptides for each sample analysed. Sample physicochemical characteristics were assessed and visualised using box and whisker plots.

Gene Ontology (GO) analysis was performed using protein annotations retrieved from Ensembl (www.ensembl.org) and GOSlim UniProtKB-GOA (www.ebi.ac.uk/GOA) with hypergeometric testing to determine GO term significance. The protein extraction buffers’ (with or without addition of PEG) protein selection bias, as well as residual proteins of the sample pellets, was assessed with regard to subcellular localisation, using GO analysis. Results from the GO annotation were visualised with a bar plot showing percentages of proteins belonging to each GO term and their location.

The percentages of missed cleavages for all samples were calculated and graphically visualised and displayed using Pandas, NumPy and Matplotlib Python packages (Python Software Foundation, Wilmington, Delaware, United States).

## Results

### Effect of polyethylene glycol on peptide and protein identification

We processed the FFPE colonic resection tumour tissues of three patients (diagnosed as indicated in Table 1). For all three patients, non-fractionated LC-MS/MS analysis showed that overall, the WCPLs extracted with 2% SDS and PEG had lower numbers of identifications at both the peptide and protein levels (validated peptides = 6840 [± 588 standard deviation {s.d.}]) and validated proteins = 2302 (± 127 s.d.) (Figure 3a and Figure 3c). On the other hand, the WCPLs extracted without PEG showed higher numbers of identifications (validated peptides = 7058 [± 649 s.d.] and validated proteins = 2314 [± 230 s.d.]) with adequate reproducibility (Figure 3a and Figure 3c). The pellet samples extracted with 4% SDS showed higher overall variability at both peptide and protein levels. However, the numbers of peptide and protein identifications were high for pellets extracted without PEG (validated peptides = 5999 [± 2176 s.d.] and validated proteins = 1893 [± 555 s.d.]) and for pellets extracted with PEG (validated peptides = 4778 [± 1764 s.d.] and validated proteins = 1564 [± 456 s.d.]) (Figure 3a and Figure 3c).

For overlap calculated from merged lists of peptide sequences, 27.1% of identified peptides were shared or overlapped between all the experimental conditions (Figure 3b). Lower percentages of unique peptides were identified for the pellets (8.7% without PEG and 3.7% with PEG), compared to the WCPLs (10.3% without PEG and 11.8% with PEG). For overlap calculated from merged lists of protein accession numbers (individual as well as protein groups), 38% of identified proteins were shared or overlapped between all the experimental conditions. Lower percentages of unique proteins were identified for the pellets (5.1% without PEG and 3.5% with PEG), compared to the WCPLs (9.4% with and without PEG).

No substantial differences were observed for the physicochemical properties of the peptides for each patient (Supplementary document** –** File S2). All experimental conditions yielded comparable relative protein abundances, indicating that protein extraction with and without PEG did not introduce a substantial observable bias with regard to proteome composition.

### Evaluation of protein physicochemical properties and GO analysis

**FIGURE 4:**
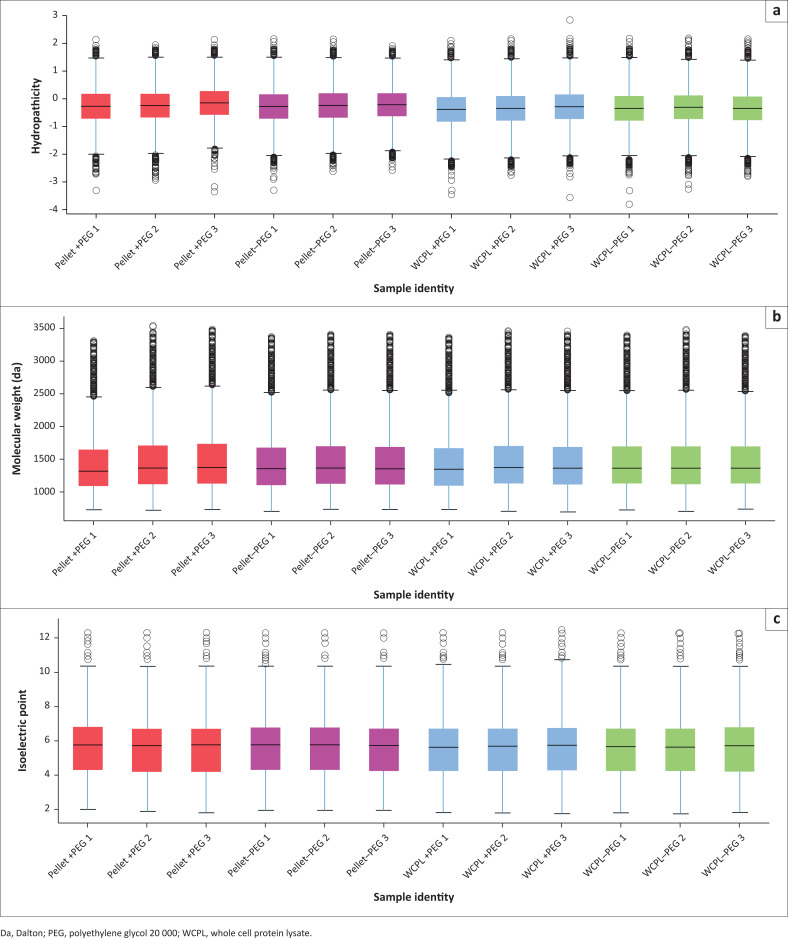
Physicochemical properties of peptides extracted under the different experimental conditions at the South African National Bioinformatics Institute, University of the Western Cape, Bellville, South Africa, from August 2017 to July 2019. (a) Hydropathicity was based on Kyte and Doolittle’s (1982) GRand AVerage of hydropathY (GRAVY) scoring matrix. (b) Molecular weight. (c) Isolectric point (pI). (–PEG) refers to protein extracted without PEG and (+PEG) refers to protein extracted with PEG. Red boxplots refer to pellet samples extracted with PEG; Purple boxplots refer to pellet samples extracted without PEG; Blue boxplots refer to WCPL samples extracted with PEG; Green boxplots refer to WCPL samples extracted without PEG.

The hydropathicity scales of all identified peptides generated from each experimental condition were similar (Figure 4a). The majority of proteins extracted (with and without PEG) and processed via the HILIC/SP3 method were hydrophilic, since the average hydropathicities of all samples were negative (in accordance with the Kyte and Doolittle scale^38^ and as described by Farias^39^). Some differences were observed between pellet samples and WCPLs (extracted with and without PEG). The pellet samples seemed slightly more hydrophobic or neutral (closer to 0) in nature compared to the WCPLs. However, neither the addition nor omission of PEG from the protein extraction buffer affected or showed a substantial hydropathicity preference or selection bias with regard to extracted peptides. The molecular weight ranges (majority > 1000 Dalton [Da] and < 2000 Da) (Figure 4b), as well as isoelectric point (pI) ranges (majority above pI 4 and below pI 7) (Figure 4c) of identified peptides were overall similar for all samples and experimental groups compared.

**FIGURE 5:**
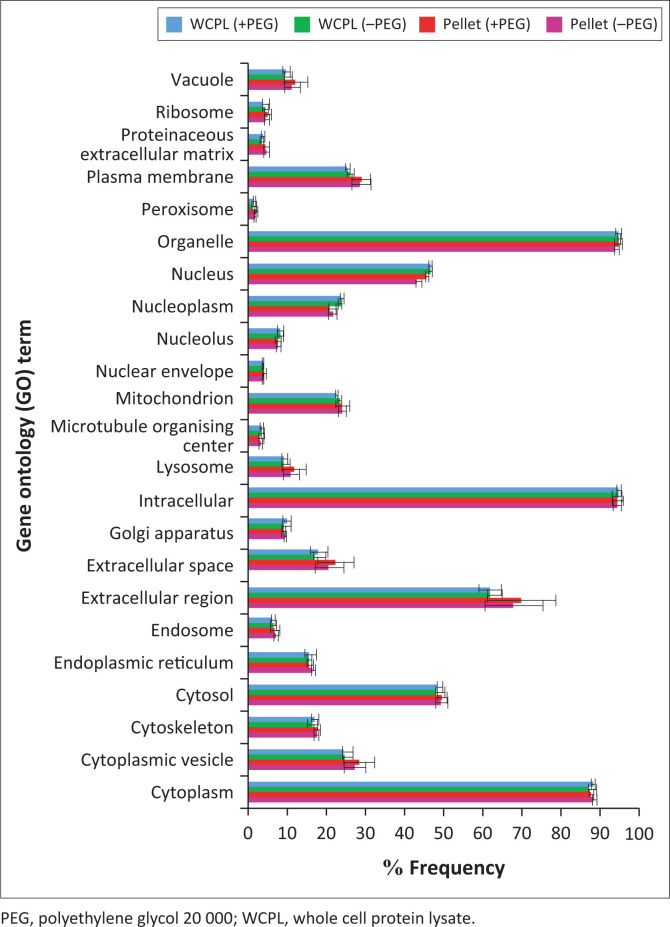
Gene ontology annotation profiles according to subcellular localisation for proteins identified from all samples and conditions at the South African National Bioinformatics Institute, University of the Western Cape, Bellville, South Africa, from August 2017 to July 2019. The average percentages of occurrence of GO terms for all three patients (per experimental group) are displayed with error bars showing standard deviation (significance level = 0.05). (–PEG) refers to protein extracted without PEG and (+PEG) refers to protein extracted with PEG. Red bar graphs refer to pellet samples extracted with PEG; Purple bar graphs refer to pellet samples extracted without PEG; Blue bar graphs refer to WCPL samples extracted with PEG; Green bar graphs refer to WCPL samples extracted without PEG.

Overall, similar GO functional annotation profiles were obtained for all samples (Figure 5). The majority of proteins were preferentially extracted from the cytoplasm (< 90%), organelles (> 90%), intracellular region (> 90%) and extracellular region (> 60%).

### Assessment of sample preparation method reproducibility and trypsin digestion efficiency

**FIGURE 6:**
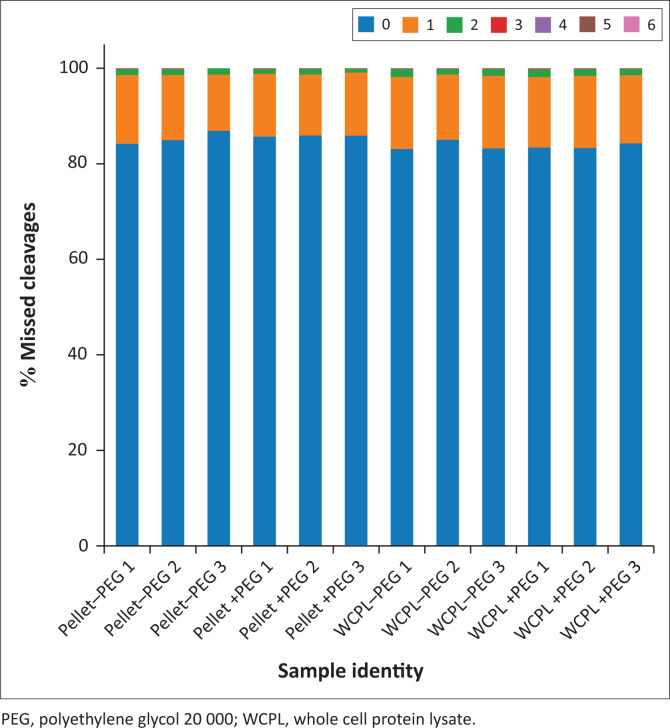
Numbers of missed cleavages for all samples at the South African National Bioinformatics Institute, University of the Western Cape, Bellville, South Africa, from August 2017 to July 2019. For each sample, the percentage of missed cleavages is shown. (–PEG) refers to protein extracted without PEG and (+PEG) refers to protein extracted with PEG.

All samples had a majority (> 80%) of fully cleaved peptides (0 missed cleavages), with approximately < 20% peptides with 1 missed cleavage, and approximately < 5% peptides with 2 missed cleavages (Figure 6). In addition, the HILIC/SP3 sample preparation method shows a similar range of missed cleavages in all samples and experimental conditions analysed.

## Discussion

In this present study, the samples processed using PEG in the protein extraction buffer had overall lower peptide and protein identifications. Using HeLa cells, Wiśniewski^13^ found that the addition of PEG to the protein extraction buffer improves protein extraction efficiency of samples that contained sub-microgram to microgram amounts of protein. However, PEG’s ability to improve protein extraction efficiency was compromised when processing cell lysates that contained more than 10 µg of protein. Furthermore, Shen^3^ found that the addition of PEG to FFPE rat tissues, which contain > 10 µg protein, failed to increase the amount of peptide and protein identifications. As our study extracted protein in the range of approximately 400 µg – 900 µg per sample (**Supplementary document –** Table S2), it would explain why PEG’s extraction efficiency was compromised and resulted in lower overall peptide and protein identifications.

The number of peptide (6840–7058) and protein (2302–2314) identifications reported here for the WCPLs fall within the range of previously published studies and are higher than those reported by Sprung^40^ (approximately 400–500 protein groups identified for triplicate samples). Craven^41^ identified between 1335 and 1945 proteins on average for four biological replicates, as well as Bronsert^4^ who identified between 3850 and 4210 peptides and between 765 and 1003 proteins on average for five biological replicates. On the other hand, Wiśniewski^13^ identified more than 6000 proteins (extracted using PEG) from the analysis of three FFPE colon cancer patient samples and they also reported higher identifications elsewhere using peptide fractionation.^22,42^ Overall, the standard deviations reported here for the WCPL samples fall within approximately 10% of the sample means. Nel^43^ reported similar or higher variances between triplicate technical replicates of a bacterial cell culture, as did Wiśniewski^13^ for human cell lines. However, sample variance and standard deviations were not explicitly reported for the aforementioned FFPE clinical sample studies.

Our results indicate that the majority of proteins were extracted in the initial WCPLs. Therefore, the extraction buffer containing 2% SDS and the extraction protocol used was sufficiently efficient to extract the majority of proteins from the patient samples; the main differences occurred due to the addition of PEG to the extraction solution. Tanca^1^ used technical replicates only for their study and found a similar variance in peptide identification overlap, ranging from as low as 26.6% for all experimental conditions to 32.6% overlap between one set of replicates from the same tissue block (patient). Our results showed similar levels of overlap between biological replicates of different tissue blocks (patients), excluding the pellet samples (which were not the main focus of the study). In addition, shared or common peptides and proteins between the pellet samples and WCPLs are due to soluble fraction or liquid (containing protein) remaining trapped within the sample pellets, after protein extraction and homogenate clarification (by centrifugation).^44^ Furthermore, the unique peptides of the pellet samples may also, in part, be attributed by the higher SDS concentration (4% SDS) used for extraction, since other studies have found greater protein extraction efficiency by using higher SDS concentrations.^4,41,42,45^

Trypsin digestion efficiency influences the molecular weight of peptides.^46^ However, all samples in this current study were subjected to the same digestion protocol. Therefore, our results show that the addition or omission of PEG to the protein extraction buffer did not affect end-result molecular weight distributions, nor were there any significant differences in molecular weight distributions of residual proteins from the pellets. Overall, neither the addition nor omission of PEG to the protein extraction solution had any selection bias with regard to extracted proteins’ physicochemical properties. Similar results were observed by Hughes^19^ and Moggridge.^47^ After processing protein extracts using the HILIC/SP3 method, they found no obvious bias with regard to the molecular mass, isoelectric point or average relative hydropathicity of resultant isolated peptides. In addition, GO analysis did not indicate any bias with regard to protein enrichment either. The HILIC/SP3 protocol also generated low percentages of missed cleavages across all samples, indicating that the workflow was sufficiently reproducible and efficient at removing any interfering chemicals (such as PEG and SDS). Batth,^48^ Hughes^49^ and Moggridge^47^ have also demonstrated the sensitivity, reproducibility and efficiency of the HILIC/SP3 sample preparation method in removing sample contaminants for optimal recovery of peptides for LC-MS/MS analysis.

### Limitations

The current study had access to tissue samples that were not limited with regard to sample volumes and concentrations required for MS analysis compared to, for example, limited samples such as fine needle biopsies. Therefore, it was neither feasible nor cost-beneficial for us to determine the effects of PEG at < 10 µg protein, since this was not compatible with the material we had available, and did not fall within the scope of the present study or studies stemming from it.^12^

### Conclusion

Using FFPE human colorectal cancer resection tissue, we demonstrated that the addition of 0.5% PEG to protein extraction buffer resulted in overall lower peptide and protein identifications, compared to buffer without the addition of PEG. In addition, protein samples extracted without PEG showed higher reproducibility, and the addition of PEG to the protein extraction buffer generated lower percentages of unique peptides remaining in the sample pellets. By expanding on previous studies that only analysed FFPE animal tissues and human cells, we have demonstrated that high protein concentrations (> 10 µg) from FFPE human colon tissue also compromises the function of PEG. The data from this study, together with our recently published selection of protein purification protocols for different FFPE block ages,^12^ should provide pathologists with an optimised methodological approach to exploit the use of archival FFPE tissue blocks.
